# Comprehensive Characterization of Bruton’s Tyrosine Kinase Inhibitor Specificity, Potency, and Biological Effects: Insights into Covalent and Noncovalent Mechanistic Signatures

**DOI:** 10.1021/acsptsci.4c00540

**Published:** 2025-03-12

**Authors:** Antonia C. Darragh, Andrew M. Hanna, Justin H. Lipner, Alastair J. King, Nicole B. Servant, Mirza Jahic

**Affiliations:** † Eurofins Discovery, 11180 Roselle Street, Suite D, San Diego, California 92121, United States; ‡ Eurofins Panlabs, 6 Research Park Drive, St. Charles, Missouri 63304, United States

**Keywords:** BTK, covalent inhibitor, noncovalent inhibitor, potency, specificity, biological effects

## Abstract

Uncovering a drug’s mechanism of action and possible adverse effects are critical components in drug discovery and development. Moreover, it provides evidence for why some drugs prove more effective than others and how to design better drugs altogether. Here, we demonstrate the utility of a high-throughput *in vitro* screening platform along with a comprehensive panel to aid in the characterization of 15 Bruton’s tyrosine kinase (BTK) inhibitors that are either approved by the FDA or presently under clinical evaluation. To compare the potency of these drugs, we measured the binding affinity of each to wild-type BTK as well as a clinically relevant resistance mutant of BTK (BTK C481S). In doing so, we discovered a considerable difference in the selectivity and potency of these BTK inhibitors to the wild-type and mutant proteins. Some of this potentially contributes to the adverse effects experienced by patients undergoing therapy using these drugs. Overall, noncovalent BTK inhibitors showed stronger potency for both the wild-type and mutant BTK when compared with that of covalent inhibitors, with the majority demonstrating a higher specificity and less off-target modulation. Additionally, we compared biological outcomes for four of these inhibitors in human cell-based models. As expected, we found different phenotypic profiles for each inhibitor. However, the two noncovalent inhibitors had fewer off-target biological effects when compared with the two covalent inhibitors. This and similar in-depth preclinical characterization of drug candidates can provide critical insights into the efficacy and mechanism of action of a compound that may affect its safety in a clinical setting.

## Graphical abstract

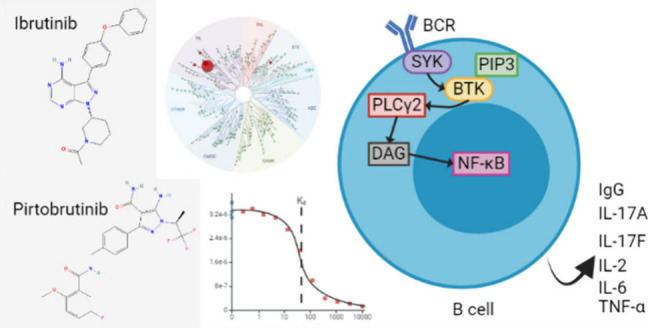

Kinases are important regulatory proteins that serve various signaling mechanisms *in vivo*.^1^ A compromised kinase, for example through over/under-activation or a mutation, may result in the progression of various diseases such as cancer^1^ and for kinases that function in the immune system, autoimmune diseases.^2^ Bruton’s tyrosine kinase (BTK) is a nonreceptor protein-tyrosine kinase in the TEC (tyrosine kinase expressed in hepatocellular carcinoma) family.^3^ BTK is downstream of the B cell receptor (BCR) signaling cascade that promotes the development of various immune cell types (e.g., ref 4). BTK is critical for B cell development and the function of mature B cells, playing a significant role in B cell malignancies (e.g., refs 5–7 ). In B cell malignancies, active BCR signaling promotes proliferation, cell survival, and resistance to apoptosis.^5,8–10^ BCR signaling is also involved in the interaction between chronic lymphocytic leukemia (CLL) cells and the tumor microenvironment.^11^ The tumor microenvironment is the complex and heterogeneous extracellular matrix surroundings of a tumor that support cancer cell survival.^12^ It includes interactions between the tumor and the innate and adaptive immune systems.^12^ The first U.S. Food and Drug Administration (FDA)-approved BTK inhibitor, ibrutinib, was found to be efficacious for treating not only B cell malignancies but also autoimmune disease.^13^ In CLL patients ibrutinib disrupts tumor-microenvironment interactions by disrupting interactions between macrophages and CLL cells in bone marrow through inhibiting secretion of the chemokine CXCL13 and decreasing CD68+ cellular extensions to CLL cells.^14^ Ibrutinib has been very successful in treating patients; however, adverse effects of the drug have also been observed (e.g., ref 15). For example, mild to moderate diarrhea, nausea, and fatigue^16,17^ and atrial fibrillation^18^ have been observed following ibrutinib treatment. Fortunately, these adverse effects are considerably less severe than those from nonspecific chemotherapy, which used to be the primary treatment for B cell malignancies.^11,19^ However, adverse effects are much less tolerated when treating immune diseases as compared with cancer.^11^ Apart from adverse events, a more common reason for the discontinuation of ibrutinib treatment is the development of resistance, often through a cysteine to serine mutation in the active site of BTK (BTK C481S).^20–23^ In addition to BTK mutations, resistance to BTK inhibitors commonly occurs in the downstream BCR signaling pathway protein, phospholipase Cγ2 (PLCG2).^24^ Another mechanism of BTK inhibitor resistance is through the tumor microenvironment, which can promote tumor growth (e.g., ref 25). Next-generation BTK inhibitors are focused on maintaining ibrutinib’s efficiency in treating B cell malignancies and/or autoimmune diseases with fewer adverse effects and less development of resistance (e.g., refs 26 and 27 ).

At least 27 BTK inhibitors have entered clinical trials, six of which have been approved for treatment in humans by at least one government agency.^11^ Acalabrutinib became the first-in-class treatment for mantle cell lymphoma (MCL) when it was approved by the FDA in 2017.^28^ Acalabrutinib significantly decreased (9.4% *v* 16.0%; P = 0.02) adverse cardiovascular events compared to ibrutinib in a phase III clinical trial (NCT02477696) in patients with CLL.^29^ Similar to ibrutinib, resistance to acalabrutinib commonly occurs through BTK C481 mutations, mostly C481S but also C481 to arginine (R) and C481 to tyrosine (Y).^30^ Acalabrutinib treatment increases apoptosis of CLL cells likely through changes in expression of the apoptotic family of Bcl-2 proteins.^31^ Acalabrutinib therapy affects the tumor microenvironment through reduced T-cell exhaustion in CLL by expanding Interferon gamma (IFN-γ)-producing CD8+ T cells.^32^ T cell expansion has not been observed following acalabrutinib treatment, as it has for ibrutinib; this is likely because acalabrutinib does not inhibit ITK like ibrutinib does.^32^ In 2023, zanubrutinib became the first-in-class treatment in the U.S. for CLL and SLL, and is now approved for the treatment of relapsed or refractory follicular lymphoma.^28^ A phase III trial (NCT03734016) comparing zanubrutinib and ibrutinib in relapsed/refractory (R/R) CLL and small lymphocytic lymphoma (SLL) found a significantly (*P* < 0.001; 78.3% *v* 62.5% after 15 months) higher overall response rate (ORR, which included partial and complete responses) and fewer cardiovascular adverse events (2.5% *v* 10.1%; two-sided P = 0.001) for zanubrutinib compared to ibrutinib.^33^ Resistance BTK mutations against zanubrutinib include C481S, C481R, leucine 528 to tryptophan (L528T), and threonine 474 to isoleucine (T474I).^34,35^ In Japan, tirabrutinib was approved by the Pharmaceuticals and Medical Devices Agency (PMDA) for the treatment of primary central nervous system diffuse large B cell lymphoma (PCNS DLBCL) in 2020.^36,37^ A three-year follow-up phase I/II study with tirabrutinib in R/R PCNSL lymphoma patients found a 63.6% overall response rate (includes 9 complete response (CR), 7 unconfirmed CR, and 12 partial response (PR) patients).^38^ Resistance to tirabrutinib commonly occurs through BTK mutations, C481S, L528W, and T474I.^39^ China’s National Medicines and Pharmaceutical Administration (NMPA) approved orelabrutinib for the treatment of MCL, CLL, and SLL in 2020.^40^ A phase I/II study (NCT03494179) for the efficacy of orelabrutinib in patients with R/R MCL found an 81.1% overall response rate (27.4% CR and 53.8% PR).^41^ In addition to BTK resistance mutations C481S, C481R, L528W, and T474I, developing in patients treated with orelabrutinib, other patients had disease progression associated with enlarged lymph nodes or increasing CLL counts in their peripheral blood.^42^ Pirtobrutinib (LOXO-305) is the first noncovalent BTK inhibitor to be granted FDA approval.^43^ It received accelerated approval in 2023 for the treatment of R/R MCL (after treatment with at least one other BTK inhibitor and another systemic therapy), as well as of CLL and SLL (after treatment with at least one other BTK inhibitor and a B-cell lymphoma-2 (BCL2) inhibitor).^43^ Pirtobrutinib treatment overcomes some BTK inhibitor resistance through its ability to bind to BTK mutants, including BTK C481S.^22,44^ Unfortunately, resistance to pirtobrutinib treatment occurs through the accumulation of other BTK mutations (e.g., at residues L528, T474, and V416) and second-site kinase-dead mutations.^22,45^ Pirtobrutinib is being tested in many active clinical trials, including a phase II trial of patients with CLL and SLL resistant to covalent BTK inhibitors (NCT06466122); the results of this and other studies will be very interesting.

Most BTK inhibitors hinder BTK activity by binding to the active site of BTK, i.e., its adenosine triphosphate (ATP)-binding pocket; this is the case for all the inhibitors used in this study (e.g., Figure 1).^44–50^ While all covalent BTK inhibitors in this study bind covalently (irreversibly) to C481, most noncovalent inhibitors rely less on interactions with this residue, and therefore some are able to inhibit BTK with mutations at the C481 site.^22^ For example, the noncovalent inhibitor fenebrutinib bound to wild-type BTK does not form any bonds with C481 but does form hydrogen bonds with methionine 477, aspartic acid 539, and lysine 430 (PDB 5VFI).^48^ Moreover, fenebrutinib potently binds to the common BTK resistance mutant C481S.^45^ Elamin and colleagues used simulations to predict which residues fenebrutinib interacts with in the C481S BTK mutant and found arginine 132 (R132) and R156 to be the most important.^51^

**Figure 1:**
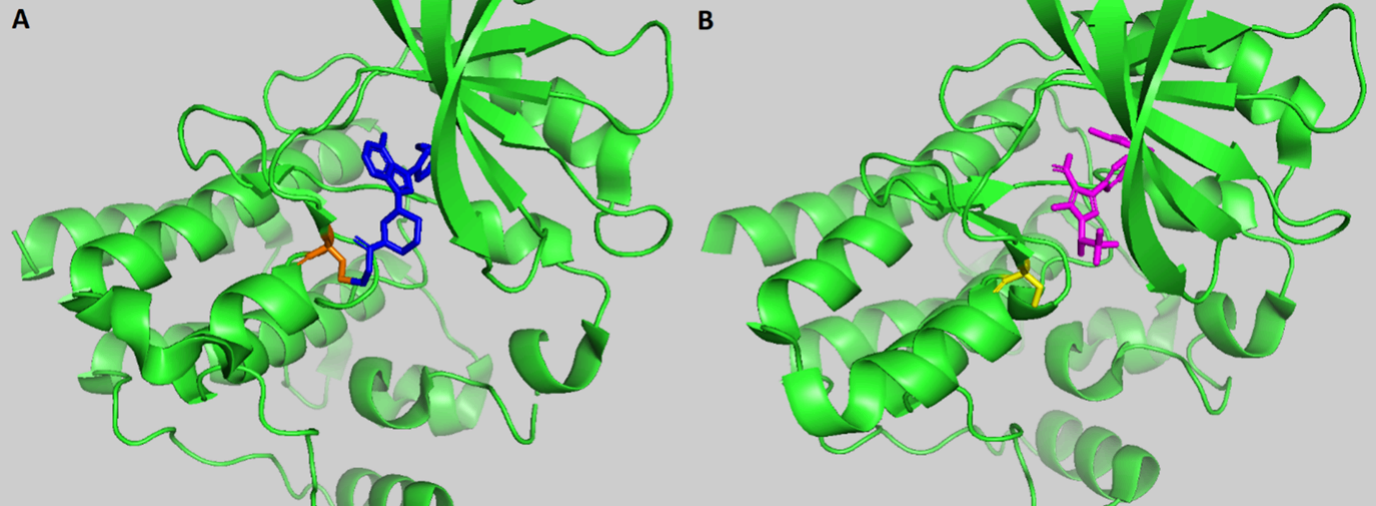
BTK inhibitors with different modes of action bound to BTK. (A) Ibrutinib, covalently bound to the ATP-binding pocket of wild-type BTK. BTK is shown in green. Cysteine 481 is highlighted in orange. Ibrutinib is shown in dark blue. (B) Pirtobrutinib, noncovalently bound to the ATP-binding pocket of mutant BTK C481S. BTK C481S is shown in green. Serine 481 is highlighted in yellow, and Pirtobrutinib is in magenta. These images were created using PyMOL from the Protein Databank (PDB) entries, 5P9J^46^ and 8FLN,^44^ respectively.

In addition to resistance mutations that change C481, 10 other kinases have a cysteine residue at an analogous location in their active site, making them likely off-targets of covalent BTK inhibitors.^47^ These kinases comprise the other four TEC family kinases (BMX, ITK, TEC, and TXK) and six other kinases (BLK, EGFR, ERBB2, ERBB4, JAK3, and MKK7).^52^ These putative off-targets are thought to contribute to some of the adverse effects of treatment with BTK inhibitors.^26,53^ For example, inhibition of the tyrosine-protein kinase TEC is associated with bleeding due to its role in platelet aggregation.^54,55^

## Results

In this study, we present an extensive collection of data on 15 BTK inhibitors that are currently FDA-approved or in clinical development. We provide resolution with respect to biochemical potency, selectivity, and biological effects of various BTK inhibitors, including the differential evaluation of covalent and noncovalent inhibitors. These insights will help determine clinical dosing and may provide mechanistic clues as to why some drugs do better in the clinic than others.

### Most Next-Generation BTK Inhibitors Are More Selective than Ibrutinib

We utilized the scanMAX Kinase Profiling Panel (Eurofins Discovery, San Diego, CA) to determine the *in vitro* ATP-independent selectivity of 15 BTK inhibitors, when tested at 1 μM, to 468 kinases/kinase domains (409 of which are wild-type sequences and 403 are from unique proteins). This panel covers approximately 75% of the human kinome. A hit was counted when at least 65% of the kinase had been competed off the control ligand (a percent of control (POC) value <35) at the given concentration.^56^ As expected, all the inhibitors tested hit BTK quite strongly (POC of 0 to 0.65) (Figure 2; Table S1). The total number of unique hits per inhibitor varied from 2 to 98 (Table 1, Figure 3). Most off-target hits observed were within the Tyrosine Kinase (TK) family (Figure 2). Remibrutinib, a covalent inhibitor, showed the most specificity, having only one off-target hit; while nemtabrutinib, a noncovalent inhibitor, showed the least specificity with 97 off-target hits (Table 1, Figure 3; Figure S1). Angst and colleagues found the same scanMAX specificity profile for 1 μM remibrutinib.^57^ We categorized the selectivity of these inhibitors as high (≤11 off-targets), moderate (12–50 off-targets), and low (>50 off-targets) (Table 1). These categories did not correlate specifically with the mode of action of these inhibitors (Table 1; Figure 3).

**Figure 2:**
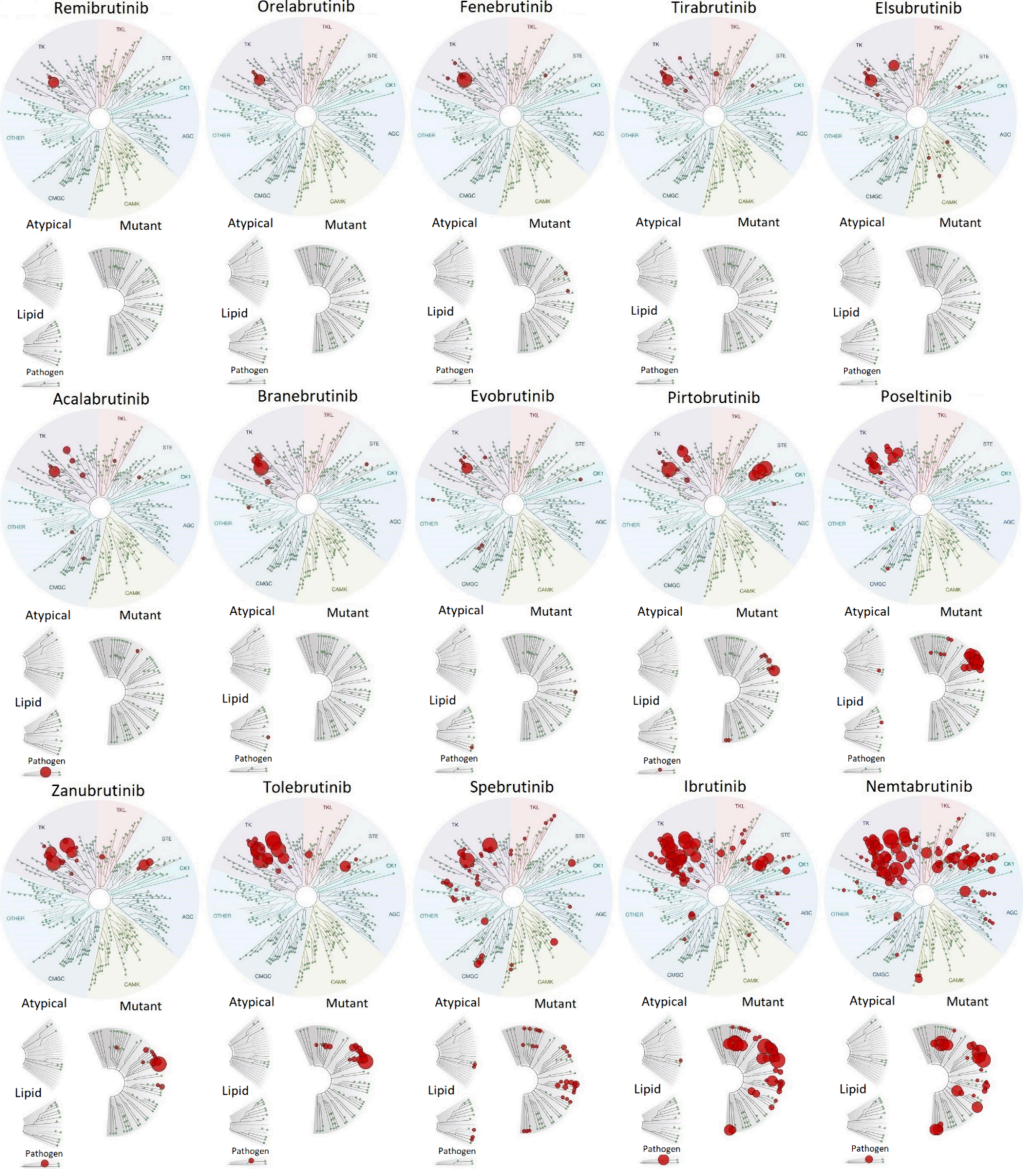
Kinome selectivity profile comparison of 15 known BTK inhibitors. Kinome selectivity results from scanMAX (Eurofins Discovery) displayed on TREEspot images. Kinases are grouped by relatedness. Kinases that a BTK inhibitor significantly (at least 65%) competed off their control ligand are shown as red dots. The five different trees/groups are wild-type, atypical, lipid, pathogen, and mutant kinases. Dot size indicates hit strength, so the larger the red dot, the stronger is the hit. The BTK inhibitors are placed in order of most selective (top left) to least selective (bottom right).

**Figure 3:**
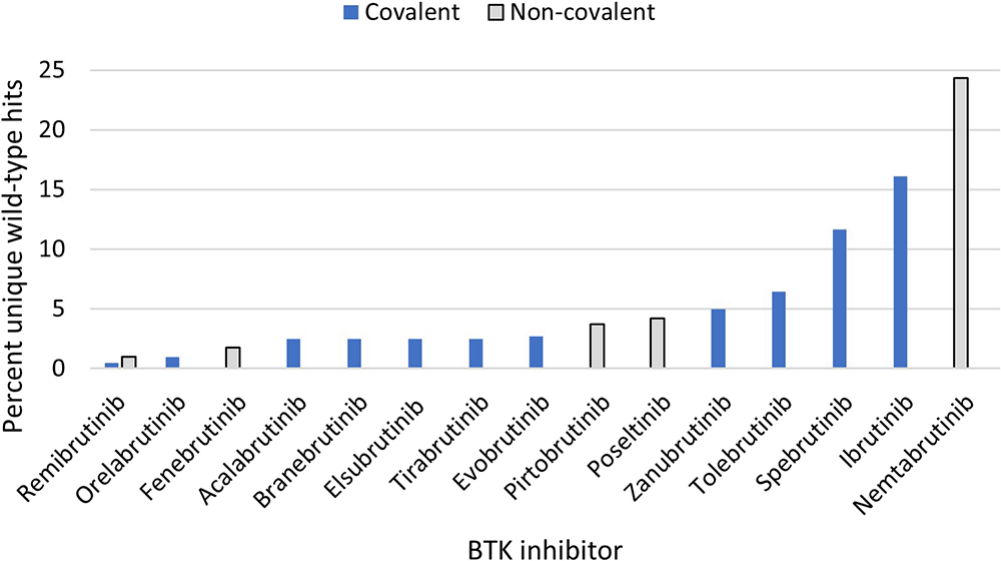
Selectivity ranking of included BTK inhibitors. The percentage of hits seen against the 403 unique wild-type kinases is plotted for each inhibitor tested. The compounds are plotted from the most specific (left) to least specific (right). Noncovalent compounds are highlighted (gray bars with black outline).

**Table 1:** Summary of Selectivity and Binding Affinity Results for 15 Known BTK Inhibitors^a^

compound name (other names)	mechanism	selectivity	number of hits (S(35))^a^	potency for BTK	*K*_d_ for wild-type BTK (nM)	potency for BTK C481S	*K*_d_ for BTK C481S (nM)
Branebrutinib (BMS-986195)	covalent	high	10	very strong	0.11	weak	430
Remibrutinib (LOU064)	covalent	high	2	very strong	0.25	weak	210
Zanubrutinib (BGB-3111, Brukinsa)	covalent	moderate	20	very strong	0.38	moderate	49
Ibrutinib (Imbruvica, PCI-32765)	covalent	low	65	very strong	0.62	strong	7.5
Pirtobrutinib (LOXO-305)	noncovalent	moderate	15	very strong	0.81	very strong	0.89
Tolebrutinib (SAR-442168, PRN2246)	covalent	moderate	26	strong	1.1	moderate	15
Fenebrutinib (GDC-0853)	noncovalent	high	7	strong	1.5	very strong	0.17
Poseltinib (LY-3337641, HM-71224)	noncovalent	moderate	17	strong	1.5	very weak	>1000
Spebrutinib (CC-292, AVL-292)	covalent	moderate	47	strong	2.3	very weak	>1000
Elsubrutinib (ABBV-105)	covalent	high	10	strong	2.9	weak	460
Acalabrutinib (Calaquence, ACP-196)	covalent	high	10	strong	4.2	weak	250
Orelabrutinib (ICP-022)	covalent	high	4	strong	4.6	very weak	>1000
Tirabrutinib (GSK-4059, ONO-4059, Velexbru)	covalent	high	10	strong	5.6	weak	250
Nemtabrutinib (ARQ 531, MK-1026)	noncovalent	low	98	strong	9.9	moderate	11
Evobrutinib (M-2951)	covalent	high	11	moderate	18	very weak	>1000

a BTK inhibitors are rank-ordered based on our measured K_d_ values for wild-type BTK.

b The selectively score (S(35)) is the number of unique kinases (out of 403) that were competed off their control ligand by at least 65% at the screening concentration of 1 μM.^56^

Our results correlate well with published results (e.g., refs 26, 58, and 59 ). For example, ibrutinib bound to the four other TEC family kinases, interleukin-2-inducible T cell kinase (ITK/TSK), TEC, bone marrow-expressed kinase (BMX/ETK), and T cell-expressed kinase (TXK/RLK)^60^ (Table S1).

Most next-generation BTK inhibitors bound to TEC and BMX with a POC similar to ibrutinib's, but did not bind significantly to ITK (e.g., refs 60 and 61 ; Table S1). Additionally, the binding of TXK by next-generation BTK inhibitors was reduced when compared with that of ibrutinib (Table S1). Moreover, ibrutinib bound more to the other kinases with a cysteine in their active site, whereas most next-generation inhibitors had less specificity for many of these kinases (Table S2). This was especially so for mitogen-activated protein kinase kinase 7 (MKK7/SEK2/JNKK2), which was only found to be a weak hit for tolebrutinib and elsubrutinib (Table S2).

### No Apparent Correlation Found between the Potency of BTK Inhibitors to Wild-Type BTK and Their Mode of Action

Using Eurofins Discovery’s KdELECT Kinase Assay Panel (San Diego, CA), we measured the ATP-independent *in vitro* binding affinity of the aforementioned BTK inhibitors to wild-type BTK. Most of these BTK inhibitors bound to BTK with single digit nanomolar affinities (Table 2). The range of affinities for BTK was 0.11 nM to 18 nM (Table 2). Branebrutinib showed the strongest affinity and evobrutinib showed the weakest affinity (Table 2). We categorized the affinity of each inhibitor into one of three groups: very strong (<1 nM), strong (1 nM to 10 nM), and moderate (10 nM to 100 nM) (Table 1). No apparent correlation between BTK binding affinity and BTK inhibitor binding mechanism was observed (Table 2).

**Table 2:** Comparison of In-House and Published BTK Inhibitor Binding Affinity to Wild-Type BTK^a^

compound name	mechanism	average in-house *K*_d_ for BTK (nM)	number of replicates (*N*)^a^	average published *K*_d_ for BTK (nM) and references	fold difference between in-house and published *K*_d_’s	average published IC_50_ for BTK (nM) and references
Branebrutinib	covalent	0.11	4	0.56 ^57^	5.1	0.1^b^ ^57,73^
Remibrutinib	covalent	0.25	4	0.63 ^57^	2.5	1.3 ^57^
Zanubrutinib	covalent	0.38	4	NDA^c^	NA^d^	0.26 ^67,70^
Ibrutinib	covalent	0.62	4	15 ^7,62,64^	1.6	1.1 ^13,45,47–49,57,59,60,62,67,70^
Pirtobrutinib	noncovalent	0.81	1	0.9 ^45^	1.1	3.2 ^27,45^
Tolebrutinib	covalent	1.1	4	NDA^c^	NA^d^	0.55 ^65^
Fenebrutinib	noncovalent	1.5	1	0.2 ^45^	7.5	2.3 ^45,48^
Poseltinib	noncovalent	1.5	1	NDA^c^	NA^d^	3.0 ^47,68^
Spebrutinib	covalent	2.3	4	NDA^c^	NA^d^	5.0 ^48,60,66,69^
Elsubrutinib	covalent	2.9	4	3.1 ^63^	1.1	180^e^ ^,^ ^63^
Acalabrutinib	covalent	4.2	4	16.2 ^57,62^	3.9	25.8 ^26,48,57,60,62^
Orelabrutinib	covalent	4.6	4	NDA^c^	NA^d^	1.6 ^71^
Tirabrutinib	covalent	5.6	4	14 ^57^	2.5	9.6 ^48,57,60^
Nemtabrutinib	noncovalent	9.9	1	46 ^45,64^	4.6	0.85 ^64^
Evobrutinib	covalent	18	4	16 ^57^	1.1	25 ^48,57,59,72^

a BTK inhibitors are rank-ordered based on our measured *K*_d_ values for wild-type BTK. References that are listed in the table:^13^,^26^,^27^,^45^,^47–49^,^57^,^59^,^60^, and^62–73^ .

b Each experiment was performed in technical duplicate (*N* = 2).

c May be at low end of detection limit in Watterson et al.^73^

d No data available.

e Not applicable.

f IC_50_ from partial binding curve.

Most of our measured K_d_ values for wild-type BTK were within 5.1-fold of published K_d_ values for these interactions (Table 2).^45,57,62–64^ The only exception is fenebrutinib, for which our measured K_d_ is 7.5-fold larger than the published K_d_.^45,48^ This slight discrepancy may be due to differences in experimental design; for example, Wang and colleagues measured the K_d_ of fenebrutinb at 4 °C whereas we measured the K_d_ at room temperature.^45^ Our fenebrutinb K_d_ is within 2-fold of the published *in vitro* half-maximal inhibitory concentration (IC_50_) value for this interaction48 (Table 2). For the five inhibitors that we did not find published K_d_s for, we compared their K_d_s to published *in vitro* IC_50_s (Table 2).^47,48,60,65–71^ They were all within 3-fold of each other (Table 2).

### Most Noncovalently Binding BTK Inhibitors Bound the Clinically Relevant Mutant BTK C481S Protein More Tightly than Did Covalently Binding BTK Inhibitors

We measured the ATP-independent *in vitro* binding affinity of these BTK inhibitors to a clinically relevant resistance mutation of BTK (BTK C481S). The range of affinities for BTK C481S was 0.17 nM to greater than 1 μM (Table 3). Fenebrutinib (GDC-0853), a noncovalent inhibitor, had the strongest affinity while evobrutinib, orelabrutinib, poseltinib, and spebrutinib (all covalent inhibitors except poseltinib) did not demonstrate any binding at the concentrations tested (Table 3). We categorized the affinity of each inhibitor into one of five groups: very strong (<1 nM), strong (1 nM to 10 nM), moderate (10 nM to 100 nM), weak (100 nM to 1 μM), and very weak (>1 μM) (Table 1).

**Table 3:** Comparison of In-House and Published BTK Inhibitor Binding Affinities for Mutant BTK C481S^a^

compound name	mechanism	average in-house *K*_d_ for BTK C481S (nM)	number of replicates (*N*)^a^	average published *K*_d_ for BTK C481S (nM) and references	fold difference between in-house and published *K*_d_	average published IC_50_ for BTK C481S (nM) and references
Fenebrutinib	noncovalent	0.17	1	5.1 ^45^	30	7.8^b^ ^45^
Pirtobrutinib	noncovalent	0.89	1	2.6 ^45^	2.9	4^b^ ^45^
Ibrutinib	covalent	7.5	1	18.1 ^45,64^	2.4	504 ^49,59^
Nemtabrutinib	noncovalent	11	1	41.8 ^45,64^	3.8	0.39 ^64^
Tolebrutinib	covalent	15	1	NDA^c^	NA^d^	NDA^c^
Zanubrutinib	covalent	49	1	69 ^45^	1.4	NDA^c^
Remibrutinib	covalent	210	1	NDA^c^	NA^d^	NDA^c^
Acalabrutinib	covalent	250	1	358 ^45^	1.4	NDA^c^
Tirabrutinib	covalent	250	1	NDA^c^	NA^d^	NDA^c^
Branebrutinib	covalent	430	1	NDA^c^	NA^d^	NDA^c^
Elsubrutinib	covalent	460	1	NDA^c^	NA^d^	NDA^c^
Evobrutinib	covalent	>1000	1	NDA^c^	NA^d^	7854 ^59^
Poseltinib	noncovalent	>1000	1	NDA^c^	NA^d^	NDA^c^
Orelabrutinib	covalent	>1000	1	NDA^c^	NA^d^	NDA^c^
Spebrutinib	covalent	>1000	1	NDA^c^	NA^d^	NDA^c^

a BTK inhibitors are rank-ordered based on our measured *K*_d_ values for mutant BTK C481S.

b Each experiment was peformed in technical duplicate (*N* = 2).

c IC_50_ measured in TMD8 cells (not *in vitro*).

d No data available.

e Not applicable.

The majority of noncovalent inhibitors tested showed stronger affinities for BTK C481S than did covalent inhibitors (Table 3). However, three covalent inhibitors, ibrutinib, tolebrutinib and zanubrutinib all had K_d_ values for BTK C481S of less than 50 nM (Table 3). We did not find many published IC_50_ or K_d_ values for this interaction but for those that are available, the majority were within 4-fold of each other (Table 3).^45,49,59,64^ The only exception to this is a 30-fold difference for fenebrutinib as compared with its published K_d_ value^45^ (Table 3).

We ranked the potency of these BTK inhibitors against wild-type BTK and BTK C481S and found that overall GDC-0853 (fenebrutinib), a noncovalent inhibitor, showed the strongest combined affinity to both proteins (Figure S2). Orelabrutinib, a covalent inhibitor, had the weakest total affinity for both proteins (Figure S2). Overall, most of the noncovalent inhibitors evaluated had stronger combined affinities for these two forms of BTK than the covalent inhibitors tested (Figure S2). This result is consistent with previously published results (e.g., refs 22, 44, and 64 ).

### Noncovalent BTK Inhibitors Displayed Fewer Nonspecific Biological Effects than Covalent Inhibitors

Testing the biological effects of a compound provides a valuable insight into the mechanism of action, most efficacious dose, and possible adverse effects that might be expected clinically.^74–76^ We compared the biological effects elicited by ibrutinib, spebrutinib, pirtobrutinib, and fenebrutinib using the BioMAP Diversity PLUS Panel (Eurofins Discovery, St. Charles, MO). This panel includes 148 biomarkers in 12 human primary cell systems that model different aspects of human tissue biology and disease. No cytotoxic effects were observed for any of these compounds at the concentrations tested (Figure 4, no black arrows). All four BTK inhibitors showed antiproliferative activity in the T cell-activated B cell model (BT) at all four concentrations tested (Figure 4, gray arrows), likely through inhibition of BTK and thus the BCR signaling pathway. This is consistent with previous results (e.g., refs 5, 14, 44, 69, and 77 ). Moreover, all compounds demonstrated a decreased secretion of the survival signals soluble interleukin-6 (sIL-6), soluble interleukin-2 (sIL-2), and soluble tumor necrosis factor alpha (sTNF-α) in the B cell model of autoimmune disease, oncology, and inflammation (Figure 4).

**Figure 4:**
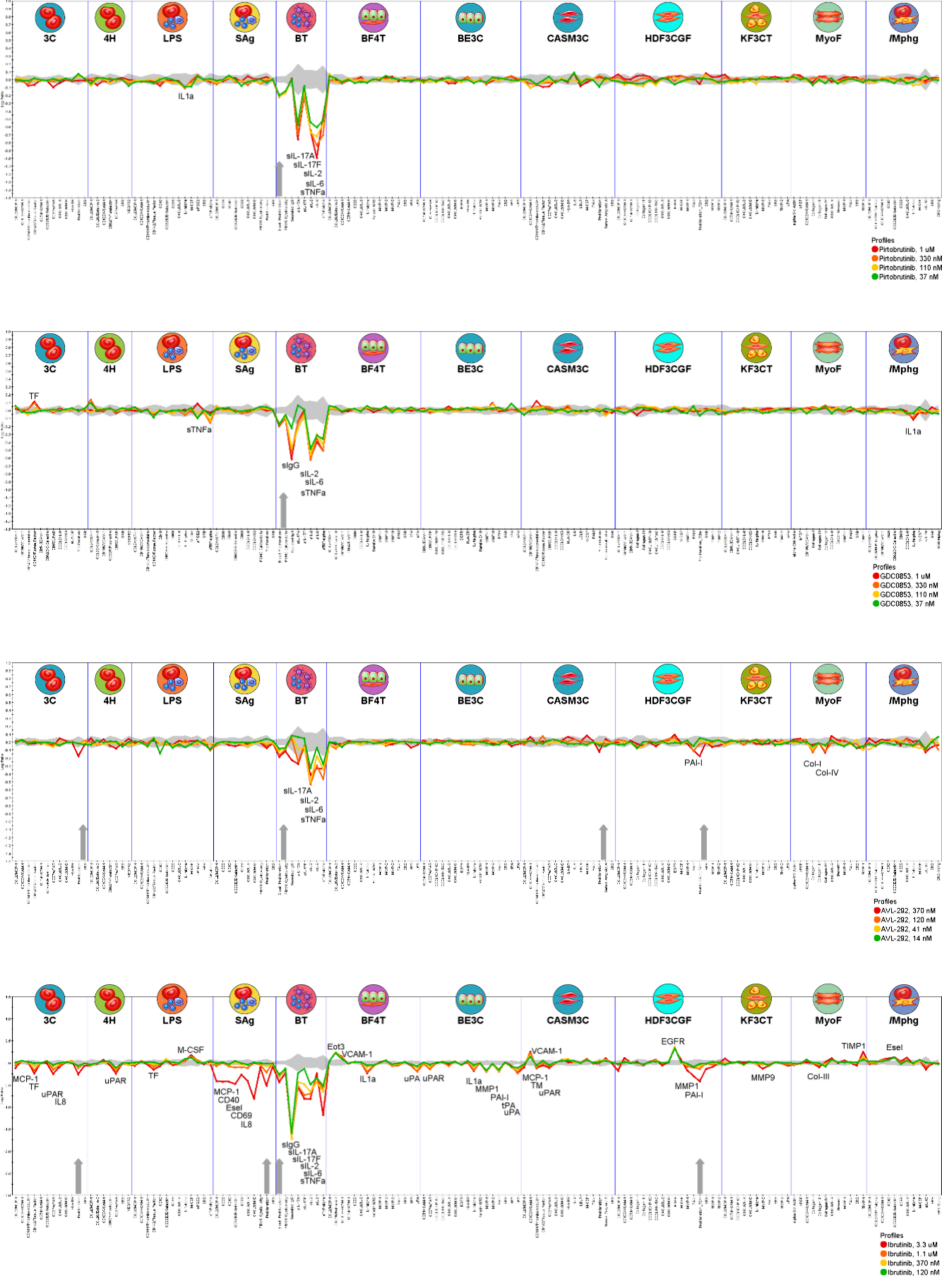
Biological effects of four BTK inhibitors in primary human cell-based models. Plots demonstrating the BioMAP Diversity PLUS Panel profile of treatment with pirtobrutinib, fenebrutinib (GDC0853), spebrutinib (AVL-292), and ibrutinib. Each compound was tested at four concentrations (indicated by red, orange, yellow, and green traces). The 12 BioMAP systems are separated by vertical lines and contain representative icons at the top. Each biomarker is listed on the *X*-axis. Annotated peaks indicate statistically significant changes in biomarker readouts when compared with the vehicle control. Changes are displayed on the *Y*-axis as a log ratio when compared with the relevant vehicle control. The 95% confidence interval for the vehicle control is shown as a shaded gray area along the zero-change midline. Gray vertical arrows on the *X*-axis signify an antiproliferative impact of the test drug at one or more concentrations; black vertical arrows (not present) would represent cytotoxic effects seen at one or more concentrations, but none were observed in this study.

Overall, the two noncovalent inhibitors that we tested had the fewest biological effects, in terms of biomarkers modulated, and those effects were the most B cell model-specific (Figure 4). Pirtobrutinib had one less effect than fenebrutinib (GDC0853) and the fewest non-B cell effects (1 of 7 effects occurred in a non-BT model versus 3 of 8 effects happened in a non-B cell model, respectively, when the inhibitors were tested at 1 μM) (Figure 4). The first-in-class BTK inhibitor, ibrutinib, showed the most biological effects (39 at 3.3 μM and 26 at 1.1 μM) and the most non-B cell model effects (32 at 3.3 μM and 19 at 1.1 μM), followed by spebrutinib (AVL-292) (11 at 370 nM, 6 of which were not in the BT model) (Figure 4).

While only minor differences were seen in inflammatory cytokine production and immune function between the noncovalent inhibitors and the second-generation covalent inhibitor (spebrutinib), a major difference observed was that treatment with spebrutinib decreased the activity of tissue remodeling biomarkers and produced antiproliferative effects in endothelial (3C), coronary artery smooth muscle (CASM), and fibroblast (HDF3CGF) cells (Figure 4, Table 4). These BioMAP results correlate well with the selectivity data generated (Figure 2), an exception being that we identified more off-targets kinases for pirtobrutinib than for fenebrutinib (Table 1), including some of the kinases with a similar cysteine residue in their ATP-binding pockets (Tables S1 and S2).

**Table 4:** Summary of Significant Biological Effects of Ibrutinib, Pirtobrutinib, Spebrutinib, and Fenebrutinib^a^

compound	concentrations (nM)	detectable cytotoxicity	antiproliferative effects	inflammation-related activities	immunomodulatory activities	tissue remodeling activities	hemostasis/angiogenesis-related activities
Ibrutinib	3300, 1100, 370, 120	none	B cells (3300, 1100, 370, 120); T cells (3300, 1100); fibroblast cells (3300, 1100); endothelial cells (3300)	↓ MCP-1, IL8, sTNFα, IL-1α; ↑ Eotaxin 3, VCAM-1; ↓↑ E-selectin	↓ CD40, CD69, sIgG, sIL-17A, sIL-17F, sIL-2, sIL-6; ↑ M-CSF	↓ uPAR, MMP1, PAI-1, MMP9, Col-III, uPA, tPA, TM; ↑ TIMP1, EGFR	↓ TF
Pirtobrutinib	1000, 330, 110, 37	none	B cells (1000, 330, 110, 37)	↓ sTNFα, IL-1α	↓ sIL-17A, sIL-17F, sIL-2, sIL-6	none	none
Spebrutinib	370, 120, 41, 14	none	B cells (370, 120, 41, 14); endothelial cells (370); CASM cells (370); fibroblast cells (370)	↓ sTNFα	↓ sIL-17A, sIL-2, sIL-6	↓ PAI-1, Col-I, Col-IV	none
Fenebrutinib	1000, 330, 110, 37	none	B cells (1000, 330, 110, 37)	↓ sTNFα, IL-1α	↓ sIgG, sIL-2, sIL-6	none	↑ TF

a ↑ signifies an increase in biomarker level relative to the vehicle control, while ↓indicates a decrease in biomarker level relative to the vehicle control, and ↓↑ is used to show when both an increase and a decrease were seen relative to vehicle control.

Most next-generation inhibitor activities overlapped with those of ibrutinib (Figure 4; Table 4) and previous work.^44,48,69^ A notable exception was the increase in the production of Tissue Factor (TF) in the 3C model (representing cardiovascular disease and chronic inflammation) following fenebrutinib treatment versus the decreased TF level seen after ibrutinib treatment in the same model (Figure 4; Table 4). Additionally, spebrutinib showed antiproliferative activity in CASM cells (modeling cardiovascular inflammation and restenosis), which was not seen with any other BTK inhibitor tested. This antiproliferative activity was not accompanied by any significant biomarker changes in this model (Figure 4). Spebrutinib also decreased the levels of different tissue remodeling biomarkers when compared with ibrutinib (i.e., collagen-I and IV versus collagen-III, respectively) (Figure 4; Table 4).

## Discussion

Accurately dissecting why drugs with the same target perform differently in clinical trials is challenging. Comparing drugs under the same conditions is a good way to tease out differences and gain a better understanding of each drug’s mechanism of action, biological outcomes, and potential adverse effects. Moreover, integration of information on specificity, potency, and biological effects early in drug discovery helps to quickly optimize therapeutic candidates. Here we measured the kinome-wide specificity and K_d_ values of 15 BTK inhibitors. We found a 96-fold difference in binding specificity, a 164-fold difference in wild-type BTK binding affinity, and a 2706-fold difference in the binding affinity for the clinically relevant mutant BTK C481S protein among the 15 BTK inhibitors (Table 1). There was not a strong correlation between the binding mechanism and specificity (Figure 3) nor binding to wild-type BTK (Table 2); however, noncovalent inhibitors generally bound more tightly to BTK C481S than covalent inhibitors (Table 3). This is consistent with clinical results, as noncovalent inhibitors have had some success in treating diseases where resistance to covalent inhibitors has developed (e.g., refs 22, 23, 27, and 64 ). Moreover, we found differences in the biological effects of three next-generation inhibitors; the noncovalent inhibitors, pirtobrutinib and fenebrutinib, primarily exhibited inflammation-related and immune-related effects, whereas the covalent inhibitor, spebrutinib, also exhibited tissue remodeling activity in non-B cell models (Figure 4; Table 4). The different phenotypic profiles of covalent and noncovalent BTK inhibitors are likely due to secondary pharmacology effects, since they all have similar B cell-specific phenotypes that are likely due to the inhibition of BTK. For example, decreases of two collagens, Col-I and Col-IV, in the MyoF BioMAP cell model (model of fibrosis, among other things) after treatment with spebrutinib (Figure 4; Table 4) may be due to direct inhibition of the three c-Jun N-terminal kinases (JNK1–3/MAPKP8–10), which we found to be unique spebrutinib scanMAX hits (Table S3). A JNK inhibitor, CC-930, was found to prevent bleomycin-induced fibrosis in TSK1 mice and decease already established fibrosis in cultured fibroblasts.^78^

Most next-generation BTK inhibitors have reduced numbers of off-target interactions when compared with ibrutinib (Figure 2). Second-generation covalent inhibitors have even less specificity to the other TEC family members and other kinases with a cysteine residue in their ATP-binding site than does ibrutinib (Tables S1 and S2), consistent with published results (e.g., ref 26). This increased specificity is not accompanied by a loss of effectiveness in targeting BTK and treating B cell malignancies (e.g., refs 29 and 79 ), which is consistent with the BioMAP human cellular phenotypic data generated in this study (Figure 4). Moreover, this increased specificity makes these compounds potentially better candidates for the treatment of autoimmune diseases.^11^

Interestingly, some of ibrutinib’s off-target effects might be beneficial for the treatment of other diseases.^37^ For example, the ability of ibrutinib to inhibit epidermal growth factor receptor (EGFR), human epidermal growth factor receptor 2 (HER2), Erb-B2 receptor tyrosine kinase 3 (ERBB3), and Erb-B2 receptor tyrosine kinase 4 (ERBB4) suggest it could be used to kill HER2+ breast cancer cells at a lower concentration than the established treatment with lapatinib.^80^ This is consistent with our selectivity results, which showed that ibrutinib binds to EGFR, ERBB3, and ERBB4 to a similar extent as it does to BTK *in vitro* (Table S2). Also, Wang and colleagues found that spebrutinib (AVL-292) did not inhibit EGFR, HER2, ERBB3, and ERBB4, a result which is consistent with our selectivity results but for one exception: we found a moderate interaction (POC of 17) between spebrutinib and ERBB4 when tested at 1 μM (Table S2). Our EGFR and JAK3 specificity results are also consistent with *in vitro* and cellular data generated for spebrutinib and acalabrutinib.^26,66^

The inhibition of multiple kinases, much like combination therapies using highly selective targeted drugs, was the idea behind the development of the noncovalent inhibitor nemtabrutinib.^64^ Consistent with this, nemtabrutinib exhibited the most off-target interactions among our selectivity results (Figure 2; Table 1). Preclinical studies of nemtabrutinib showed promising results in mice models.^64^ Reiff and colleagues identified an increase in survival in the Eμ-TCL1 engraftment model of CLL and an Eμ-MYC/TCL1 engraftment model resembling Richter’s transformation in nemtabrutinib- versus ibrutinib-treated mice. It will be interesting to compare the effectiveness of nemtabrutinib with dual BTK inhibitors such as QL-X-138, a BTK/MNK (Mitogen-Activated Protein Kinase Interacting Kinase),^81^ in clinical trials.

BTK combination therapies have shown potential for improving lymphoma treatment.^82^ BTK inhibitors ibrutinib (e.g., ref 83), acalabrutinib (NCT03946878), and zanubrutinib (NCT03336333) have been or are being tested in combination with the BCL2 inhibitor venetoclax to treat CLL/SLL and/or MCL.^82^ BCL2 is an antiapoptotic protein that if improperly regulated can contribute to B cell lymphomas.^84^ These BTK inhibitors and ventoclax are all efficient a monotherapies but dual treatment may have synergistic benefits. The results of ongoing randomized trials comparing the effects of monotherapies and combinations of these drugs will be very intriguing.

Another promising dual-target therapy is BTK degraders. These drugs work through ubiquitination and proteasomal degradation of their target(s). Some have emerged as promising treatment options for patients who have already received treatment with a covalent and/or noncovalent BTK inhibitors and a BCL2 inhibitor.^85,86^ This includes patients with BTK mutations of residues C481, L528, T474, and V416.^86^ The BTK degrader, NX-2127–001, not only degrades BTK but also the transcription factor IKAROS family zinc finger 3 (IKZF3).^86^ It would be interesting to test if dual treatment with a BTK inhibitor and an IKZF3 inhibitor would work similarly.

We found that the strongest biological effects produced by the four BTK inhibitors tested were on B cells and their microenvironments (Figure 4). This is expected when considering the function of BTK in B cells and published results (e.g., refs 5, 44, 69, and 77 ). Additionally, spebrutinib’s effect on the level of sIL-17A produced, but not that of sIL-17F, is consistent with findings that these cytokines are regulated independently in B cells^87^ (Figure 4). Treatment with all four BTK inhibitors produced decreased levels of cytokines sIL-6, sIL-2, and sTNF-α (Figure 4; Table 4). The decreases of sIL-6 and sTNF-α, tumor-promoting cytokines, would likely inhibit the tumor microenvironment.^88^ However, counter to this, the decrease of sIL-2, an antitumor response activator, would likely be beneficial for the tumor microenvironment.^89^ It would be interesting to investigate if these responses essentially cancel each other out. We were surprised to find that ibrutinib treatment increased production of EGFR in the HDF3CGF model (representing wound healing and inflammation) (Figure 4). Prior evidence (e.g., refs 90 and 91 ) and our selectivity results (Table S2) support direct inhibition of EGFR by ibrutinib, thus we expected a decrease in activity. However, ibrutinib treatment was also antiproliferative in this HDF3CGF model and decreased levels of the tissue remodelers MMP1 and PAI-I suggested that this increase in EGFR was not causing significant proliferative effects. It may also be that the increase in EGFR seen here represented the activation of a feedback loop in the cells to try and compensate for EGFR inhibition by the BTK inhibitor treatment. Another interesting finding is that all four BTK inhibitors tested decreased the production of TNF-α, a target for rheumatoid arthritis (RA), in the B cell model (Figure 4). This is not surprising since BTK is known to regulate TNF-α in RA (e.g., ref 92). What was somewhat unexpected is that fenebrutinib, but not spebrutinib, has shown significant efficacy in treating RA patients.^69,93^ However, the spebrutinib phase IIa clinical trial (NCT01975610) did show statistically significant reductions in inflammatory markers compared to placebo.^69^ Additionally, this study had a smaller sample size and duration that may have underscored the efficacy of spebrutinib in RA.^69,94^ Moreover, both ibrutinib and fenebrutinib block platelet aggregation from Fc-receptor CD32a (FcγRIIA) activation, which occurs in heparin-induced thrombocytopenia type II (HIT).^95^ Activated tissue factor (TF) expression is involved in the development of this condition; therefore, it is somewhat surprising that we found opposing effects of ibrutinib and fenebrutinib on TF activity in the 3C Th1 vasculature BioMAP system (Figure 4; Table 4). This suggests that these BTK inhibitors may have slightly different effects with respect to this condition, possibly through off-target effects or due to their different modes of action. Another consideration is that the effects of ibrutinib and fenebrutinib on TF levels were only seen at the higher concentrations of these inhibitors tested, whereas Goldmann and colleagues found that a low concentration of these inhibitors is sufficient for blocking platelet aggregation and thus making them potential candidates for HIT treatment.^95^

Even though BTK inhibitors were the primary focus of this study, the screening cascade and various technologies we utilized may be applied to other target classes and chemical matter in a similar fashion. These preclinical tools thus provide the ability to conduct in-depth head-to-head comparisons of drug candidates, yielding valuable insights into a drug’s engagement with its intended target and off-targets that could affect a broad range of biological effects, which may ultimately affect both efficacy and safety in the clinical therapeutic setting.

## Methods

### Small Molecules

Ibrutinib, acalabrutinib, tirabrutinib, relabrutinib, zanubrutinib, pirtobrutinib, nemtabrutinib, fenebrutinib, oseltinib, spebrutinib, branebrutinib, evobrutinib, elsubrutinib, olebrutinib, and remibrutinib were purchased from MedChemExpress (Monmouth Junction, NJ).

### Protein Constructs and Protein Expression

Full length wild-type BTK and mutant BTK C481S constructs were fused N-terminally with the DNA binding domain of NFκB (consisting of residues 35–36 fused to residues 41–359 (as described in ref 96), using UniProt entry P19838 as a reference) and expressed in transiently transfected HEK293 cells. Protein extracts were harvested in M-PER extraction buffer (Pierce Biotechnology, Rockford, IL) supplemented with 150 mM NaCl, 10 mM Dithiothreitol (DTT), Protease Inhibitor Cocktail Complete (Roche Diagnostics GmbH, Mannheim, Germany), and Phosphatase Inhibitor Cocktail Set II (Merck KGaA, Darmstadt, Germany) following the manufacturer’s guidelines. The fusion protein was labeled with a DNA tag containing the NFκB binding site fused to an amplicon for qPCR readout, which was added directly to the expression extract.

### Competition Binding Assays

Small molecule binding to wild-type and mutant kinases was assessed using ATP site-dependent competition binding assays as previously described.^56,97–99^ Streptavidin-coated magnetic beads (Thermo Fisher Scientific, Waltham, MA) were incubated with a biotinylated affinity ligand for 30 min at 25 °C. Liganded beads were blocked with excess biotin (125 nM) and washed with a blocking buffer containing SeaBlock (Pierce Biotechnology), 1% Bovine Serum Albumin (BSA) and 0.05% Tween 20. The binding reactions were prepared with the DNA-tagged BTK protein extract, ligated affinity beads, and the competitor test compounds in a binding buffer (1x Phosphate Buffered Saline (PBS), 0.05% Tween 20, 10 mM DTT, 0.1% BSA, 2 mg/mL sheared salmon sperm DNA) in deep well, natural polypropylene 384-well plates (catalog number 784201, Greiner Bio-One, Kremsmünster, Austria) in a final volume of 19.7 μL. These test compounds were prepared as 111x stocks in 100% Dimethyl sulfoxide (DMSO). All compounds for K_d_ measurements were distributed by acoustic transfer (noncontact dispensing) in 100% DMSO. The compounds were diluted directly into the assays such that the final concentration of DMSO was 0.9%. No enzyme purification steps were performed on the protein extracts before adding them to the reaction mixture, and the protein extracts were diluted 10,000-fold in the final reaction mixture (the final DNA-tagged enzyme concentration was less than 0.1 nM). Binding assay mixtures were incubated at 25 °C with shaking for 1 h. Then affinity beads were extensively treated with a wash buffer (1x PBS, 0.05% Tween 20) to remove unbound protein from the protein lysate. Using an elution buffer (1x PBS, 0.05% Tween 20 and 0.5 μM affinity ligand) the beads were resuspended and incubated at 25 °C while shaking for a 30 min period. The concentration of wild-type or mutant BTK in the eluates was then determined using quantitative PCR. K_d_ values for each competitor compound were determined using 11 serial 3-fold dilutions and three DMSO control points. Each assay’s affinity ligand concentration on the magnetic beads has been optimized to ensure that the true thermodynamic K_d_ values for competitor molecules were measured, as described in detail previously99.

### Data Analysis for Competitive Binding Assays

K_d_s were calculated using a standard dose–response curve fitting of the data using the Hill equation: Response = Background + (Signal – Background)/(1 + (K_d_^Hill Slope^/Dose^Hill Slope^). The Hill Slope was set to −1. A nonlinear least-square fit using the Levenberg–Marquardt algorithm was employed for curve fitting.

### Human Primary Cell Systems

BioMAP Diversity PLUS Panel (Eurofins Discovery, St. Charles, MO) is composed of 12 human primary cell cultures or cocultures. The systems and stimuli have been described previously.^100^ Adherent cells were added to 96-well plates, allowed to reach confluence, and incubated for 1 h with the indicated BTK inhibitor or assay control, all dissolved in 0.1% DMSO. The cells were then stimulated with cytokines, agonists, or growth factors and cultured for a minimum of 24 h at 37 °C in 5% CO_2_. Assay controls included DMSO-only (vehicle control), nonstimulated conditions (negative control), and a positive control test agent (colchicine at 1 μM).

### Measured Biomarkers

Following the system-specific incubation time, a total of 148 protein biomarkers spanning all 12 cell systems were measured, either in cellular extracts by direct ELISA, or in supernatants by bead-based multiplex immunoassays, homogeneous time-resolved fluorescence (HTRF) detection or capture ELISA. Cell proliferation and viability were measured using 0.1% sulforhodamine B (SRB, Sigma–Aldrich) after fixation with 10% TCA, and reading wells at 560 nm for adherent cells, and using alamarBlue reduction (Bio-Rad) for suspension cells. These biomarkers were previously described by Shah and colleagues.^100^

### Biomarker Analysis

For each biomarker, the value obtained from the BTK inhibitor-treated samples was divided by the average of 8 DMSO-only controls from the same plate, yielding a ratio that was then transformed to the log_10_ scale. Data from historical controls of prior BioMAP studies were used to define a range (95% confidence interval) for each ratio called an “envelope.” Experimental ratios that decreased more than 20% (<0.097 on the log_10_ scale) or increased more than 20% (>0.079 on the log_10_ scale) when compared with the envelope were considered significant, and annotated when observed for 2 or more consecutive concentrations. For cytotoxicity, we considered a decrease in the SRB readout greater than 50% (<−0.3 on the log_10_ scale) significant and excluded from the analysis the corresponding concentrations. The acceptance criteria and quality assurance of the BioMAP experiments have been previously described.^100^

## Supplementary Material



## Notes

(1) Comparison of BTK inhibitor specificity to TEC family members; (2) Kinase profile comparison of most and least selective BTK inhibitors; (3) Comparison of BTK inhibitor specificity to human epidermal growth factor receptor 2+ (HER2+) associated kinases; (4) Potency curves of 15 BTK inhibitors to BTK and mutant BTK (BTK C481S); (5) Comparison of BTK inhibitor specificity to c-Jun N-terminal kinases (JNK1-3/MAPKP8-10) (PDF)
