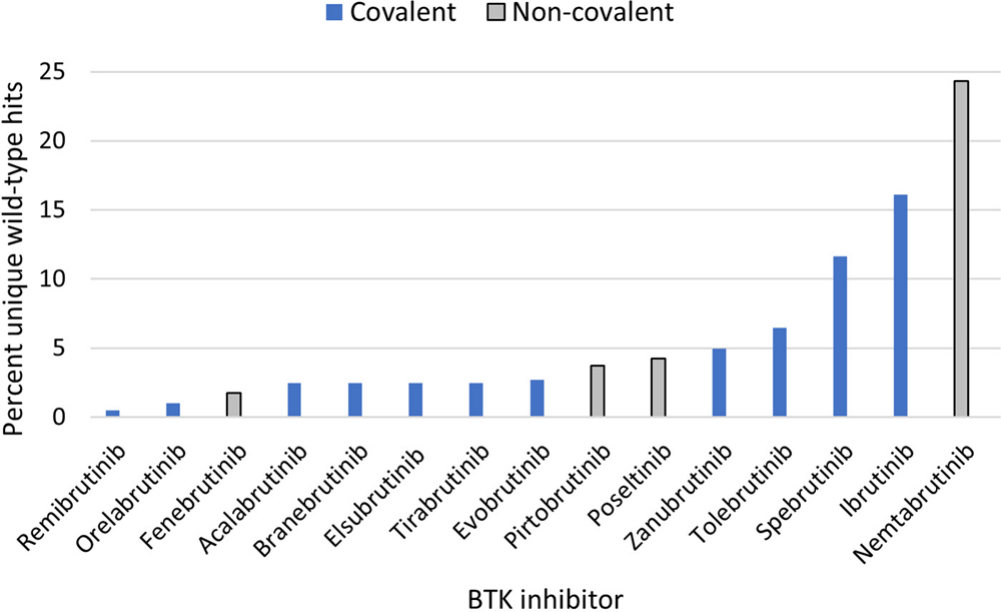# Correction to “Comprehensive Characterization of Bruton’s Tyrosine Kinase Inhibitor Specificity, Potency, and Biological Effects: Insights into Covalent and Noncovalent Mechanistic Signatures”

**DOI:** 10.1021/acsptsci.5c00551

**Published:** 2026-01-09

**Authors:** Antonia C. Darragh, Andrew M. Hanna, Justin Lipner, Alastair J. King, Nicole B. Servant, Mirza Jahic

Description of correction being made to the manuscript file: We would like to correct Figure 3. There should only be a blue bar in the graph for remibrutinib (there should not be an additional gray bar with a black outline for remibrutinib). The corrected figure is below.