# Correction to “Comprehensive Characterization of Bruton’s Tyrosine Kinase Inhibitor Specificity, Potency, and Biological Effects: Insights into Covalent and Noncovalent Mechanistic Signatures”

**DOI:** 10.1021/acsptsci.6c00190

**Published:** 2026-04-21

**Authors:** Antonia C. Darragh, Andrew M. Hanna, Justin H. Lipner, Alastair J. King, Nicole B. Servant, Mirza Jahic

**Description of correction being made to the manuscript file:** We misclassified poseltinib as a noncovalent inhibitor, but it is a covalent inhibitor. We would like to correct this classification in Tables 1, 2, and 3 (by removing “non” from poseltinibs mechanism). We would also like to rephrase the results section header “**Most** Noncovalently Binding BTK Inhibitors Bound the Clinically Relevant Mutant BTK C481S Protein More Tightly than Did Covalently Binding BTK Inhibitors” to “**All** Noncovalently Binding BTK Inhibitors **Tested** Bound the Clinically Relevant Mutant BTK C481S Protein More Tightly than Did Covalently Binding BTK Inhibitors”. We would also like to remove “except poseltinib” from the text of this section. We would like to rephrase the first sentence of the second paragraph of this section “**The majority of** noncovalent inhibitors tested showed stronger affinities for BTK C481S than did covalent inhibitors (Table 3).” to “**All** noncovalent inhibitors tested showed stronger affinities for BTK C481S than did covalent inhibitors (Table 3).” The corrected tables are below.

**Table 1:** 

Compound Name (other names)	Mechanism	Selectivity	Number of hits (S(35))^a^	Potency for BTK	K_d_ for wild-type BTK (nM)	Potency for BTK C481S	K_d_ forBTK C481S (nM)
Branebrutinib (BMS-986195)	covalent	high	10	Very strong	0.11	Weak	430
Remibrutinib (LOU064)	covalent	high	2	Very strong	0.25	Weak	210
Zanubrutinib (BGB-3111, Brukinsa)	covalent	moderate	20	Very strong	0.38	Moderate	49
Ibrutinib (Imbruvica, PCI-32765)	covalent	low	65	Very strong	0.62	Strong	7.5
Pirtobrutinib (LOXO-305)	noncovalent	moderate	15	Very strong	0.81	Very strong	0.89
Tolebrutinib (SAR-442168, PRN2246)	covalent	moderate	26	Strong	1.1	Moderate	15
Fenebrutinib (GDC-0853)	noncovalent	high	7	Strong	1.5	Very strong	0.17
Poseltinib (LY-3337641, HM-71224)	covalent	moderate	17	Strong	1.5	Very weak	>1000
Spebrutinib (CC-292, AVL-292)	covalent	moderate	47	Strong	2.3	Very weak	>1000
Elsubrutinib (ABBV-105)	covalent	high	10	Strong	2.9	Weak	460
Acalabrutinib (Calaquence, ACP-196)	covalent	high	10	Strong	4.2	Weak	250
Orelabrutinib (ICP-022)	covalent	high	4	Strong	4.6	Very weak	>1000
Tirabrutinib (GSK-4059, ONO-4059, Velexbru)	covalent	high	10	Strong	5.6	Weak	250
Nemtabrutinib (ARQ 531, MK-1026)	noncovalent	low	98	Strong	9.9	Moderate	11
Evobrutinib (M-2951)	covalent	high	11	Moderate	18	Very weak	>1000

**Table 2:** 

Compound Name	Mechanism	Average in-house K_d_ for BTK (nM)	Number of Replicates (N)^a^	Average Published K_d_ for BTK (nM) and References	Fold Difference Between in-house and Published K_d_s	Average Published IC_50_ for BTK (nM) and References
Branebrutinib	covalent	0.11	4	0.56^57^	5.1	0.1^*b 57,73*^
Remibrutinib	covalent	0.25	4	0.63^57^	2.5	1.3^57^
Zanubrutinib	covalent	0.38	4	NDA^*c*^	NA^*d*^	0.26^67,70^
Ibrutinib	covalent	0.62	4	1^57,62,64^	1.6	1.1^*13,45,47–49,57,59,60,62,67,70*^
Pirtobrutinib	noncovalent	0.81	1	0.9^45^	1.1	3.2^27,45^
Tolebrutinib	covalent	1.1	4	NDA^*c*^	NA^*d*^	0.55^65^
Fenebrutinib	noncovalent	1.5	1	0.2^45^	7.5	2.3^45,48^
Poseltinib	covalent	1.5	1	NDA^*c*^	NA^*d*^	3.0^47,68^
Spebrutinib	covalent	2.3	4	NDA^*c*^	NA^*d*^	5.0^48,60,66,69^
Elsubrutinib	covalent	2.9	4	3.1^63^	1.1	180^*e 63*^
Acalabrutinib	covalent	4.2	4	16.2^57,62^	3.9	25.8^26,48,57,60,62^
Orelabrutinib	covalent	4.6	4	NDA^*c*^	NA^*d*^	1.6^71^
Tirabrutinib	covalent	5.6	4	14^57^	2.5	9.6^48,57,60^
Nemtabrutinib	noncovalent	9.9	1	46^45,64^	4.6	0.85^64^
Evobrutinib	covalent	18	4	16^57^	1.1	25^48,57,59,72^

**Table 3:** 

Compound Name	Mechanism	Average in-house K_d_ for BTK C481S (nM)	Number of Replicates (N)^a^	Average Published K_d_ for BTK C481S (nM) and References	Fold Difference Between in-house and Published K_d_	Average Published IC_50_ for BTK C481S (nM) and References
Fenebrutinib	noncovalent	0.17	1	5.1^45^	30	7.8^*b 45*^
Pirtobrutinib	noncovalent	0.89	1	2.6^45^	2.9	4^*b 45*^
Ibrutinib	covalent	7.5	1	18.1^45,64^	2.4	504^49,59^
Nemtabrutinib	noncovalent	11	1	41.8^45,64^	3.8	0.39^64^
Tolebrutinib	covalent	15	1	NDA^*c*^	NA^*d*^	NDA^*c*^
Zanubrutinib	covalent	49	1	69^45^	1.4	NDA^*c*^
Remibrutinib	covalent	210	1	NDA^*c*^	NA^*d*^	NDA^*c*^
Acalabrutinib	covalent	250	1	358^45^	1.4	NDA^*c*^
Tirabrutinib	covalent	250	1	NDA^*c*^	NA^*d*^	NDA^*c*^
Branebrutinib	covalent	430	1	NDA^*c*^	NA^*d*^	NDA^*c*^
Elsubrutinib	covalent	460	1	NDA^*c*^	NA^*d*^	NDA^*c*^
Evobrutinib	covalent	>1000	1	NDA^*c*^	NA^*d*^	7854^59^
Poseltinib	covalent	>1000	1	NDA^*c*^	NA^*d*^	NDA^*c*^
Orelabrutinib	covalent	>1000	1	NDA^*c*^	NA^*d*^	NDA^*c*^
Spebrutinib	covalent	>1000	1	NDA^*c*^	NA^*d*^	NDA^*c*^

## Supplementary Material



## Notes

Corrected Supporting Tables S1 and S2 (PDF)

