# Supplementary material for: Comprehensive Characterization of Bruton’s Tyrosine Kinase Inhibitor Specificity, Potency, and Biological Effects: Insights into Covalent and Noncovalent Mechanistic Signatures
Source: ACS Pharmacol Transl Sci. 2025 Mar 12;8(4):917–31. doi: 10.1021/acsptsci.4c00540 (PMC11997881; doi:10.1021/acsptsci.4c00540)
Supplement: Supplementary file 1 [file pt4c00540_si_001.pdf]

Supporting Information

Comprehensive Characterization of Bruton's Tyrosine Kinase Inhibitor Specificity, Potency, and Biological Effects: Insights into Covalent and Non-Covalent Mechanistic Signatures

Antonia C. Darragh[1], Andrew M. Hanna[1], Justin H. Lipner[2], Alastair J. King[2], Nicole B. Servant[1], Mirza Jahic[1]*

[1]Eurofins Discovery, 11180 Roselle Street, Suite D, San Diego, CA 92121, United States of America

[2]Eurofins Panlabs, 6 Research Park Drive, St. Charles, MO 63304, United States of America

*To whom correspondence should be addressed: Mirza Jahic, e-mail address: Mirza.Jahic@discovery.eurofinsus.com

Supporting Table S1. Comparison of BTK inhibitor specificity to TEC family members. TEC family members are Burton's tyrosine kinase (BTK), tyrosine kinase expressed in hepatocellular carcinoma (TEC), bone marrow-expressed kinase (BMX/ETK), and T cell-expressed kinase (TXK/RLK), and interleukin-2-inducible T cell kinase (ITK/TSK).

Compound Name	Mechanism	BTK binding POC[a]	TEC binding POC[a]	BMX binding POC[a]	TXK binding POC[a]	ITK binding POC[a]
Ibrutinib	covalent	0	5.9	6.4	0.5	3.6
Poseltinib	non-covalent	0.1	8.5	12	4.4	1
Branebrutinib	covalent	0	7.8	11	2	17
Spebrutinib	covalent	0	8.1	10	4.5	51
Tolebrutinib	covalent	0	4.2	11	1.1	67
Zanubrutinib	covalent	0	8.7	10	2.2	74
Nemtabrutinib	non-covalent	0.6	7.3	8.5	2.7	86
Fenebrutinib	non-covalent	0	17	16	53	25
Tirabrutinib	covalent	0.35	9.4	16	14	100
Evobrutinib	covalent	0.65	10	13	23	95
Elsubrutinib	covalent	0.1	18	20	25	94
Orelabrutinib	covalent	0.35	12	27	34	100
Pirtobrutinib	non-covalent	0	12	51	11	100
Acalabrutinib	covalent	0.2	14	52	47	100
Remibrutinib	covalent	0.15	20	47	97	97

[a] The percent of control (POC) is the percent of kinase that was competed off its control ligand relative to the positive and negative controls at the screening concentration[56]. A zero POC signifies that the compound competition was comparable to that of the positive control.

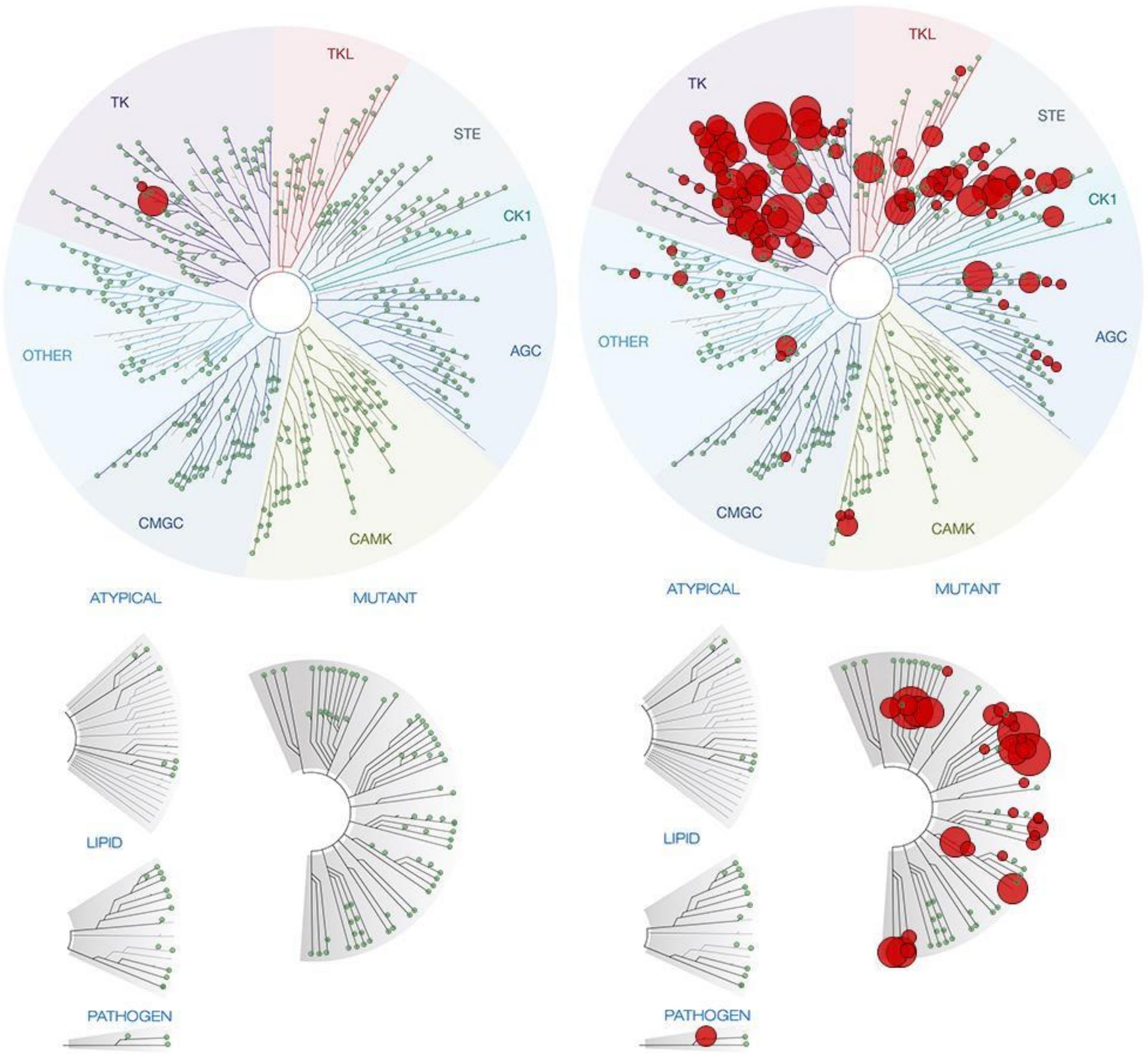

Supporting Figure S1. Kinase profile comparison of most and least selective BTK inhibitors. TREEspot™ images showcasing the selectivity kinase panel hit profile for remibrutinib (on the left), the most selective, and nemtabrutinib (on the right), the least selective, as red dots on a phylogeny of kinases. The phylogenies are of wild-type, atypical, lipid, pathogen, and mutant kinases. Dot size indicates hit strength, so the larger the red dot, the stronger the hit.

Supporting Table S2. Comparison of BTK inhibitor specificity to human epidermal growth factor receptor 2+ (HER2+)-associated kinases.

Compound Name	Mechanism	BLK[a] binding POC[b]	EGFR[c] binding POC[b]	ERBB2[d] binding POC[b]	ERBB3[d] binding POC[b]	ERBB4[d] binding POC[b]	JAK3[e] binding POC[b]	MKK7[f] binding POC[b]
Ibrutinib	covalent	0.25	0	0.1	0.15	0	0.7	0.15
Tolebrutinib	covalent	0.05	0	0	0.8	0	36	19
Nemtabrutinib	non-covalent	0.3	0	0	0.9	0.35	86	39
Zanubrutinib	covalent	0.3	0	7.3	23	0.75	20	55
Poseltinib	non-covalent	0.85	1.2	8.7	97	0.15	0.45	100
Pirtobrutinib	non-covalent	69	0.5	5.9	97	2	77	91
Acalabrutinib	covalent	57	61	4.7	64	6.9	80	75
Spebrutinib	covalent	22	49	57	83	17	0	79
Elsubrutinib	covalent	15	90	73	74	91	0.15	24
Tirabrutinib	covalent	14	74	26	12	85	73	87
Branebrutinib	covalent	3.2	77	100	75	75	48	100
Evobrutinib	covalent	22	88	91	97	32	100	100
Orelabrutinib	covalent	52	68	100	88	96	89	100
Fenebrutinib	non-covalent	60	95	97	78	100	95	89
Remibrutinib	covalent	100	99	100	100	100	100	100

[a] B lymphocyte kinase (BLK).

[b] The percent of control (POC) is the percent of kinase that was competed off its control ligand relative to the positive and negative controls at the screening concentration[56]. A zero POC signifies that the compound competition was comparable to that of the positive control.

[c] Epidermal growth factor receptor (EGFR).

[d] Erb-B2 receptor tyrosine kinases 2, 3, and 4 (ERBB2, ERBB3, and ERBB4, respectively).

[e] Janus kinase 3 (JAK3). The JAK3 POC is for its JH1 catalytic domain.

[f] Mitogen-activated protein kinase kinase 7 (MKK7).

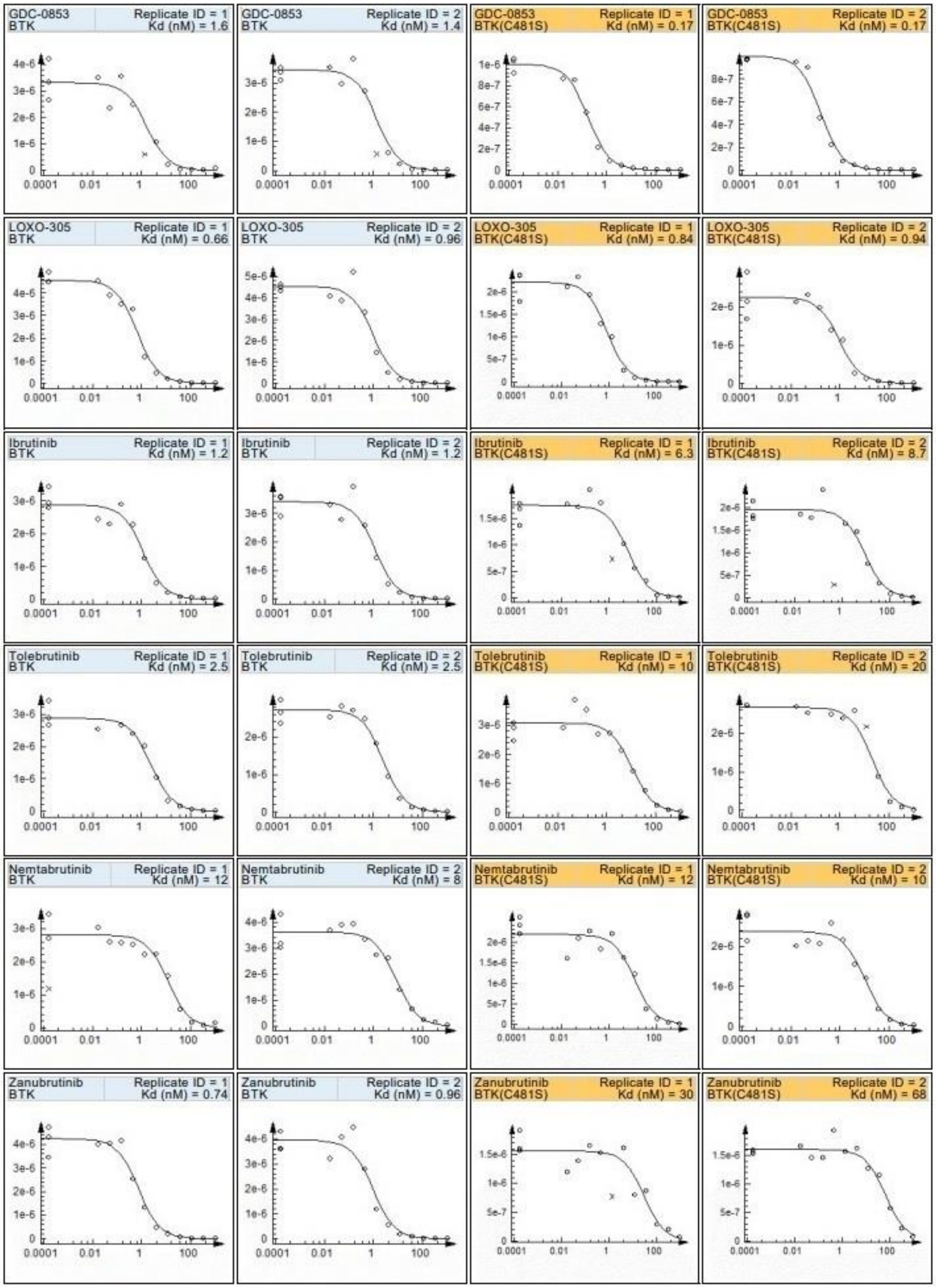
GDC-0853 BTK Replicate ID = 1 Kd (nM) = 1.6
GDC-0853 BTK Replicate ID = 2 Kd (nM) = 1.4
GDC-0853 BTK(C481S) Replicate ID = 1 Kd (nM) = 0.17
GDC-0853 BTK(C481S) Replicate ID = 2 Kd (nM) = 0.17
LOXO-305 BTK Replicate ID = 1 Kd (nM) = 0.66
LOXO-305 BTK Replicate ID = 2 Kd (nM) = 0.96
LOXO-305 BTK(C481S) Replicate ID = 1 Kd (nM) = 0.84
LOXO-305 BTK(C481S) Replicate ID = 2 Kd (nM) = 0.94
Ibrutinib BTK Replicate ID = 1 Kd (nM) = 1.2
Ibrutinib BTK Replicate ID = 2 Kd (nM) = 1.2
Ibrutinib BTK(C481S) Replicate ID = 1 Kd (nM) = 6.3
Ibrutinib BTK(C481S) Replicate ID = 2 Kd (nM) = 8.7
Tolebrutinib BTK Replicate ID = 1 Kd (nM) = 2.5
Tolebrutinib BTK Replicate ID = 2 Kd (nM) = 2.5
Tolebrutinib BTK(C481S) Replicate ID = 1 Kd (nM) = 10
Tolebrutinib BTK(C481S) Replicate ID = 2 Kd (nM) = 20
Nemtabrutinib BTK Replicate ID = 1 Kd (nM) = 12
Nemtabrutinib BTK Replicate ID = 2 Kd (nM) = 8
Nemtabrutinib BTK(C481S) Replicate ID = 1 Kd (nM) = 12
Nemtabrutinib BTK(C481S) Replicate ID = 2 Kd (nM) = 10
Zanubrutinib BTK Replicate ID = 1 Kd (nM) = 0.74
Zanubrutinib BTK Replicate ID = 2 Kd (nM) = 0.96
Zanubrutinib BTK(C481S) Replicate ID = 1 Kd (nM) = 30
Zanubrutinib BTK(C481S) Replicate ID = 2 Kd (nM) = 68

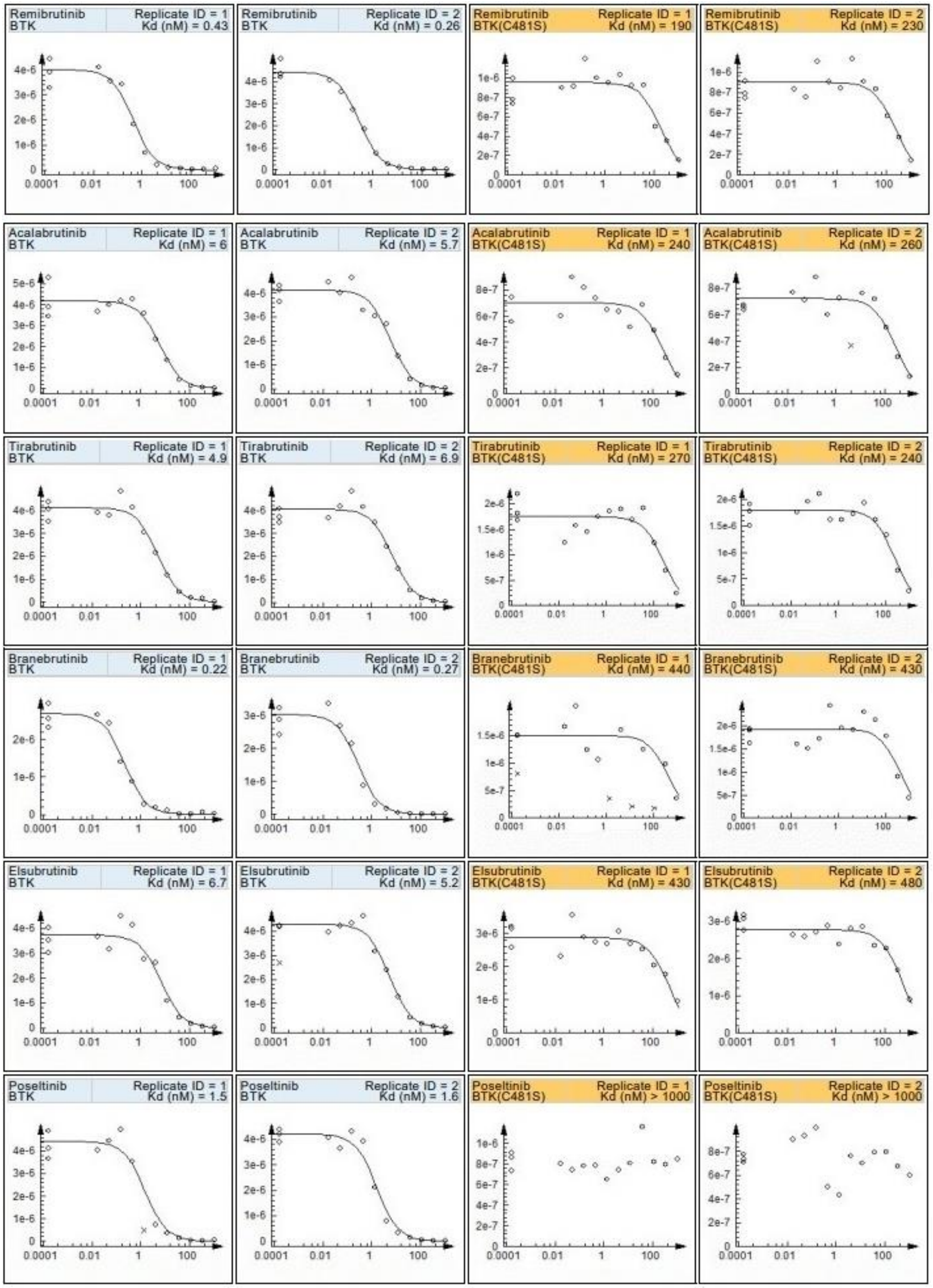
Remibrutinib BTK Replicate ID = 1 Kd (nM) = 0.43
Remibrutinib BTK Replicate ID = 2 Kd (nM) = 0.26
Remibrutinib BTK(C481S) Replicate ID = 1 Kd (nM) = 190
Remibrutinib BTK(C481S) Replicate ID = 2 Kd (nM) = 230
Acalabrutinib BTK Replicate ID = 1 Kd (nM) = 6
Acalabrutinib BTK Replicate ID = 2 Kd (nM) = 5.7
Acalabrutinib BTK(C481S) Replicate ID = 1 Kd (nM) = 240
Acalabrutinib BTK(C481S) Replicate ID = 2 Kd (nM) = 260
Tirabrutinib BTK Replicate ID = 1 Kd (nM) = 4.9
Tirabrutinib BTK Replicate ID = 2 Kd (nM) = 6.9
Tirabrutinib BTK(C481S) Replicate ID = 1 Kd (nM) = 270
Tirabrutinib BTK(C481S) Replicate ID = 2 Kd (nM) = 240
Branebrutinib BTK Replicate ID = 1 Kd (nM) = 0.22
Branebrutinib BTK Replicate ID = 2 Kd (nM) = 0.27
Branebrutinib BTK(C481S) Replicate ID = 1 Kd (nM) = 440
Branebrutinib BTK(C481S) Replicate ID = 2 Kd (nM) = 430
Elsubrutinib BTK Replicate ID = 1 Kd (nM) = 6.7
Elsubrutinib BTK Replicate ID = 2 Kd (nM) = 5.2
Elsubrutinib BTK(C481S) Replicate ID = 1 Kd (nM) = 430
Elsubrutinib BTK(C481S) Replicate ID = 2 Kd (nM) = 480
Poseltinib BTK Replicate ID = 1 Kd (nM) = 1.5
Poseltinib BTK Replicate ID = 2 Kd (nM) = 1.6
Poseltinib BTK(C481S) Replicate ID = 1 Kd (nM) > 1000
Poseltinib BTK(C481S) Replicate ID = 2 Kd (nM) > 1000

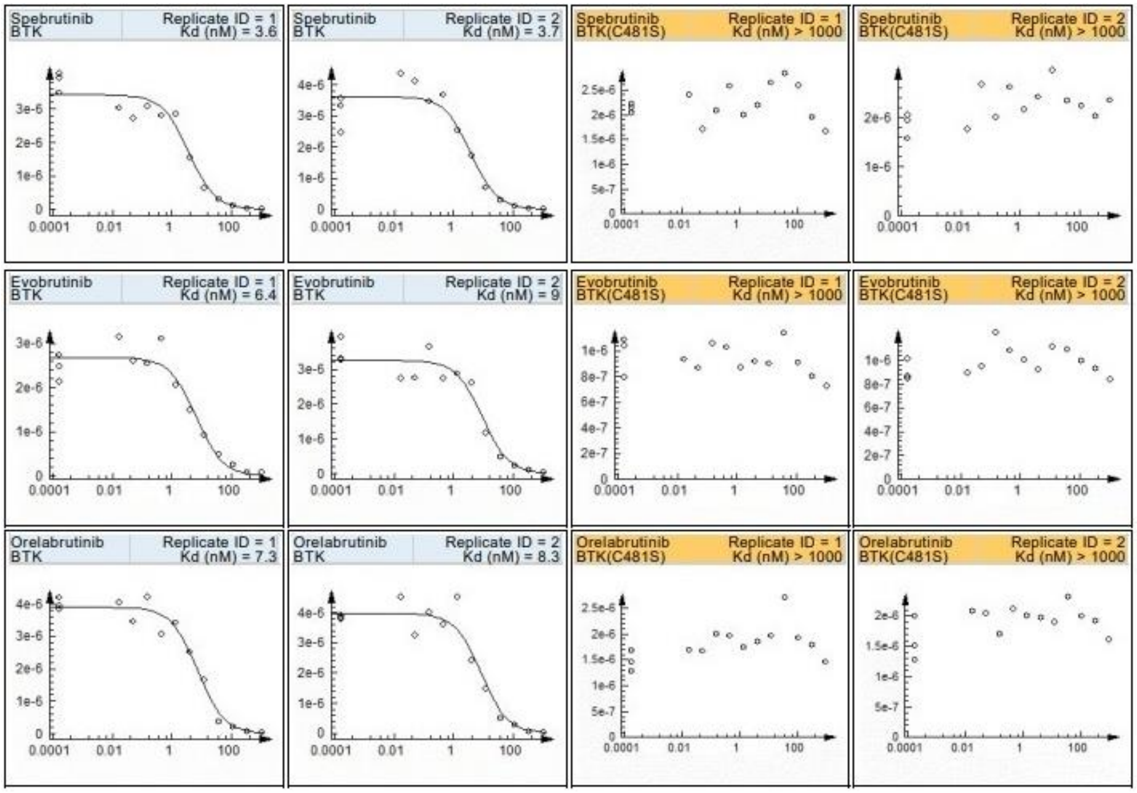

Supporting Figure S2. Potency curves of 15 BTK inhibitors to BTK and mutant BTK (BTK C481S). 11-point dose response curves (or lack thereof) for each BTK inhibitor to wild-type BTK and a BTK mutant (BTK C481S). These curves were used to determine the binding affinities (Kds) of these interactions. The BTK inhibitors are listed in order of most potent to both BTK and BTK C481S (top) to least potent (bottom). Outlier data points marked with an “x” were not used for Kd determination. Each y-axis scales with the highest measured signal for that replicate. The x-axis depicts the concentration of BTK inhibitor in units of nanomolar (nM).

Supporting Table S3. Comparison of BTK inhibitor specificity to c-Jun N-terminal kinases (JNK1-3/MAPKP8-10).

Compound Name	JNK1 (MAPK8) POC[a]	JNK2 (MAPK9) POC[a]	JNK3 (MAPK10) POC[a]
Spebrutinib	4.8	8.4	3.9
Poseltinib	32	100	100
Nemtabrutinib	39	100	100
Acalabrutinib	93	58	97
Ibrutinib	78	78	74
Branebrutinib	82	89	89
Tolebrutinib	96	83	90
Elsubrutinib	96	91	94
Tirabrutinib	99	92	96
Zanubrutinib	99	95	93
Remibrutinib	93	99	100
Pirtobrutinib	96	94	100
Evobrutinib	95	100	100
Fenebrutinib	100	100	100
Orelabrutinib	100	100	100

[a] The percent of control (POC) is the percent of kinase that was competed off its control ligand relative to the positive and negative controls at the screening concentration[56]. A zero POC signifies that the compound competition was comparable to that of the positive control.
