# Supplementary material for: Correction to “Comprehensive Characterization of Bruton’s Tyrosine Kinase Inhibitor Specificity, Potency, and Biological Effects: Insights into Covalent and Noncovalent Mechanistic Signatures”
Source: ACS Pharmacol Transl Sci. 2026 Apr 21;9(5):1238–40. doi: 10.1021/acsptsci.6c00190 (PMC13162048; doi:10.1021/acsptsci.6c00190)
Supplement: Supplementary file 1 [file pt6c00190_si_001.pdf]

Correction to “Comprehensive Characterization of Bruton’s Tyrosine Kinase Inhibitor Specificity, Potency, and Biological Effects: Insights into Covalent and Noncovalent Mechanistic Signatures” **Supporting Information**

Author names

Darragh, Antonia C

Hanna, Andrew M

Lipner, Justin

King, Alastair J

Servant, Nicole B.

Jahic, Mirza

Journal Citation

Darragh AC, Hanna AM, Lipner JH, King AJ, Servant NB, Jahic M. Comprehensive Characterization of Bruton's Tyrosine Kinase Inhibitor Specificity, Potency, and Biological Effects: Insights into Covalent and Noncovalent Mechanistic Signatures. ACS Pharmacol Transl Sci. 2025 Mar 12;8(4):917-931. doi: 10.1021/acsptsci.4c00540. PMID: 40242575; PMCID: PMC11997881.

Description of correction being made to the Supporting Information file: We misclassified poseltinib as a noncovalent inhibitor, but it is a covalent inhibitor. We would like to correct this classification in Supporting Tables S1 and S2 (by removing “non” from poseltinibs mechanism). The corrected tables are below.

Supporting Table S1

Compound Name	Mechanism	BTK binding POC[a]	TEC binding POC[a]	BMX binding POC[a]	TXK binding POC[a]	ITK binding POC[a]
Ibrutinib	covalent	0	5.9	6.4	0.5	3.6
Poseltinib	covalent	0.1	8.5	12	4.4	1
Branebrutinib	covalent	0	7.8	11	2	17
Spebrutinib	covalent	0	8.1	10	4.5	51
Tolebrutinib	covalent	0	4.2	11	1.1	67
Zanubrutinib	covalent	0	8.7	10	2.2	74
Nemtabrutinib	non-covalent	0.6	7.3	8.5	2.7	86
Fenebrutinib	non-covalent	0	17	16	53	25
Tirabrutinib	covalent	0.35	9.4	16	14	100
Evobrutinib	covalent	0.65	10	13	23	95
Elsubrutinib	covalent	0.1	18	20	25	94
Orelabrutinib	covalent	0.35	12	27	34	100
Pirtobrutinib	non-covalent	0	12	51	11	100
Acalabrutinib	covalent	0.2	14	52	47	100
Remibrutinib	covalent	0.15	20	47	97	97

Supporting Table S2

Compound Name	Mechanism	BLK[a] binding POC[b]	EGFR[c] binding POC[b]	ERBB2[d] binding POC[b]	ERBB3[d] binding POC[b]	ERBB4[d] binding POC[b]	JAK3[e] binding POC[b]	MKK7[f] binding POC[b]
Ibrutinib	covalent	0.25	0	0.1	0.15	0	0.7	0.15
Tolebrutinib	covalent	0.05	0	0	0.8	0	36	19
Nemtabrutinib	non-covalent	0.3	0	0	0.9	0.35	86	39
Zanubrutinib	covalent	0.3	0	7.3	23	0.75	20	55
Poseltinib	covalent	0.85	1.2	8.7	97	0.15	0.45	100
Pirtobrutinib	non-covalent	69	0.5	5.9	97	2	77	91
Acalabrutinib	covalent	57	61	4.7	64	6.9	80	75
Spebrutinib	covalent	22	49	57	83	17	0	79
Elsubrutinib	covalent	15	90	73	74	91	0.15	24
Tirabrutinib	covalent	14	74	26	12	85	73	87
Branebrutinib	covalent	3.2	77	100	75	75	48	100
Evobrutinib	covalent	22	88	91	97	32	100	100
Orelabrutinib	covalent	52	68	100	88	96	89	100
Fenebrutinib	non-covalent	60	95	97	78	100	95	89
Remibrutinib	covalent	100	99	100	100	100	100	100
